# Tertiary lymphoid structures in genitourinary cancers: a comprehensive review

**DOI:** 10.3389/fonc.2026.1787200

**Published:** 2026-02-25

**Authors:** Alvaro Abreu, Alejandra Viera Plasencia, Mercy Iribarren, Carter Wegner, Joana Nuraj, Elai Davicioni, Hisham F. Bahmad, Mohammed Shahait

**Affiliations:** 1 Herbert Wertheim College of Medicine, Florida International University, Miami, FL, United States; 2 Veracyte, Inc., South San Francisco, CA, United States; 3 Department of Pathology and Laboratory Medicine, University of Miami Miller School of Medicine, Miami, FL, United States; 4 Department of Urology, University of California, Irvine, Irvine, CA, United States

**Keywords:** bladder cancer, genitourinary cancers, kidney tumors, penile cancer, prostate cancer, review, tertiary lymphoid structures, testicular cancer

## Abstract

Tertiary lymphoid structures (TLSs) are lymphoid cell clusters that form in non-lymphoid tissues in response to chronic inflammation and function as sites for localized, antigen-specific immune responses, potentially enhancing anti-tumor immunity. This review examines TLS presence, composition, and clinical significance across genitourinary (GU) cancers to evaluate their potential as prognostic and therapeutic targets. In prostate cancer, TLSs are infrequently found due to a typically immunologically inactive tumor microenvironment (TME), but when present, they correlate with improved outcomes and reduced recurrence, especially when structurally mature with active germinal centers (GCs). Bladder cancer, in contrast, demonstrates increased TLS activity, particularly in high-grade disease, with high TLS density associated with superior responses to Bacillus Calmette-Guérin (BCG) therapy and anti-PD-L1 treatment. In testicular seminomas, TLSs have been associated with a more favorable prognosis, whereas non-seminomatous germ cell tumors demonstrate TLS suppression driven by SERPINB9-mediated downregulation of chemokines that promote their development. In clear cell renal cell carcinoma (ccRCC), TLSs correlate with improved survival and enhanced immunotherapy responses, although elevated *CXCL13* expression may paradoxically signal more aggressive disease. Unlike ccRCC, TLSs are infrequent in papillary and chromophobe RCC, reflecting a less-inflamed TME that likely contributes to reduced immunotherapy responsiveness and prognostic value. TLSs in penile squamous cell carcinomas show enhanced immune infiltration and improved overall survival (OS) independent of stage. Notably, mature TLSs are key for effective anti-tumor immunity, whereas immature TLSs may fail to generate an adequate response. Collectively, these findings highlight TLSs as prognostic biomarkers with prognostic value and therapeutic potential in GU malignancies.

## 1 Introduction

Tertiary lymphoid structures (TLSs) are organized lymphoid cell clusters that resemble secondary lymphoid organs (SLOs) and form in non-lymphoid tissues in response to chronic inflammation. These structures are seen in conditions such as autoimmune diseases, persistent infections, and cancer. They lack a fibrous capsule, which enables them to integrate directly into the local tissue microenvironment. At a cellular level, TLSs are composed of B-cell follicles, T-cell zones, dendritic cells, follicular dendritic cells, and high endothelial venules (HEVs), forming a microscopic niche capable of supporting antigen presentation, lymphocyte activation, and germinal center (GC) reactions (1). These features allow TLSs to function as sites for the generation of localized, antigen-specific immune responses, potentially enhancing anti-tumor immunity (2).

TLSs are dynamic structures whose development and maturation are influenced by cellular signals that promote lymphocyte aggregation and organization, driving the differentiation of early TLSs into functionally mature structures. In recent years, there has been increasing interest in these structures and their potential use as biomarkers for both prognosis and therapeutic responsiveness, particularly in the context of immune checkpoint blockade therapies (2, 3).

TLSs have been associated with increased infiltration of CD8+ T cells, memory T cells, B cells, and mature dendritic cells (4). Within the TME, these cells contribute to a coordinated anti-tumor immune response which often results in a more favorable clinical prognosis (3). Meta-analyses across various malignancy subtypes have demonstrated that the presence of TLSs correlates with improved survival outcomes, particularly in non-small cell lung cancer, colorectal cancer, and melanoma (2, 4).

The predictive value of TLSs is becoming increasingly recognized in genitourinary (GU) malignancies. In bladder cancer, TLSs have been identified in superficial and deep tumor compartments, with their composition and maturation states directly influencing immune checkpoint blockade responses (3). In prostate cancer (PCa), TLS formation is less frequent, but, if present, they play an important role in modulating immune activity within the TME. While immunotherapy has shown limited efficacy in PCa compared to bladder or kidney malignancies, new data suggest that the presence of TLS might serve as a biomarker for identifying the patient subsets that are more likely to benefit from such treatments (5). In renal malignancies, especially in clear cell renal cell carcinoma (ccRCC), TLSs have been linked to the presence of tumor-reactive T-cells and better therapeutic outcomes (2, 6). In testicular germ cell tumors, TLS-like aggregates have been documented, often in association with seminomas exhibiting strong lymphoid infiltration (1). Lastly, penile squamous cell carcinomas occasionally harbor TLSs in peritumoral regions, although their functional impact remains poorly understood (1).

**Figure 1:**
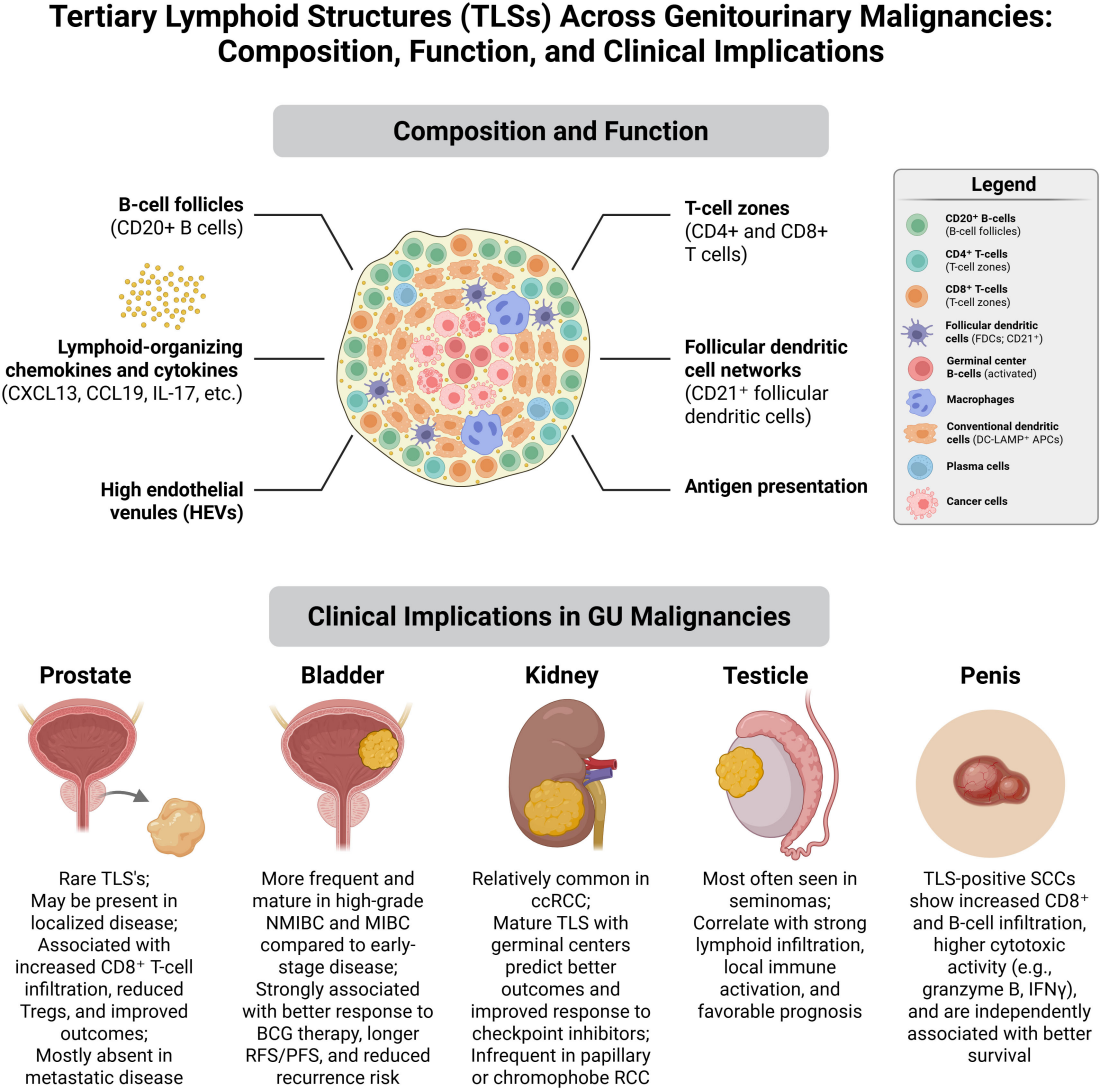
Tertiary lymphoid structures (TLSs) in genitourinary malignancies. TLSs are composed of B-cell follicles, T-cell zones, follicular dendritic cells, and HEVs, orchestrated by chemokines such as CXCL13 and CCL19 to support local immune activation. Their presence varies across GU tumors: rare but prognostically favorable in prostate cancer, frequent and predictive of BCG/ICI response in bladder cancer, common and outcome-modifying in clear cell RCC, enriched in seminomas, and survival-associated in penile SCC. The schematic was created in BioRender. Bahmad, H. (2026) https://BioRender.com/865wuzs. BCG, Bacillus Calmette-Guérin; ccRCC, clear cell renal cell carcinoma; CD, cluster of differentiation; HEV, high endothelial venule; ICI, immune checkpoint inhibitor; IFNγ, interferon gamma; IL-17, interleukin 17; MIBC, muscle-invasive bladder cancer; NMIBC, non-muscle invasive bladder cancer; PFS, progression-free survival; RCC, renal cell carcinoma; RFS, recurrence-free survival; SCC, squamous cell carcinoma; TLS, tertiary lymphoid structure.

Overall, TLSs are emerging as important immunological players in solid tumors, with growing potential as prognostic biomarkers and therapeutic targets in cancer immunotherapy. Their presence in GU tumors, though variable, may reveal key aspects of tumor immunity to guide and develop new treatments (**Figure 1**).

## 2 TLS maturity stages and immunophenotypic markers

TLS maturity is classified into three distinct stages based on specific immunophenotypic markers and architectural features. The most widely accepted classification system defines these stages as: Stage 1 (Lymphoid Aggregates), Stage 2 (Non-GC TLS), and Stage 3 (GC-like TLS) (7).

### 2.1 Stage 1: lymphoid aggregates

Lymphoid aggregates represent the earliest and most immature form of TLSs. These structures are characterized by CD20+ B-cell clusters that lack follicular dendritic cell networks (CD21-) and absence of CD23 expression (CD23-). The immunophenotypic profile is CD20+CD21-CD23-, indicating neither architectural organization nor GC functionality. They consist of loosely organized B and T lymphocytes without defined compartmentalization or specialized stromal support structures.

### 2.2 Stage 2: non-GC TLSs

Non-GC TLSs demonstrate intermediate maturation with organized architecture but lack functional GC activity. The defining immunophenotypic signature is CD20+CD21+CD23-. The presence of CD21+ follicular dendritic cells here indicates structural organization with formation of follicular dendritic cell networks, but the absence of CD23 confirms lack of GC functionality. These structures exhibit spatial segregation of B-cell and T-cell zones but do not support active GC reactions including somatic hypermutation and affinity maturation.

### 2.3 Stage 3: GC-like TLS

GC-like TLS represent the most mature, functionally active form with complete GC organization and functionality. The immunophenotypic profile is CD20+ CD21+ CD23 +. These structures contain CD23+ follicular dendritic cells within well-developed GCs, indicating full functional capacity for B-cell selection and antibody affinity maturation. Additional GC markers include BCL6+ proliferating B cells (a transcription factor essential for GC formation), Ki67+ cells (marking active proliferation with distinct dark and light zones), and BCL2- GC B cells (distinguishing them from naïve B cells).

### 2.4 Methodological approaches to TLS assessment

TLS assessment employs multiple complementary methodologies, each with distinct advantages and limitations. H&E-based identification remains the foundation of TLS detection, relying on recognition of dense lymphoid aggregates with or without visible GCs (8, 9). This approach enables initial screening but requires validation with immunohistochemistry (IHC) for accurate maturation staging. Standard IHC panels typically include CD20 (B cells), CD3 (T cells), CD21 (follicular dendritic cells), CD23 (GC marker), BCL6 (GC B cells), and Ki-67 (proliferation marker) (9, 10). A streamlined approach using H&E staining combined with CD23 IHC has demonstrated high interrater agreement (kappa 0.90) for maturation assessment and can be applied across all specimen types (9).

Quantification approaches vary substantially across studies, contributing to heterogeneity in prognostic comparisons. Common metrics include TLS density (TLS per mm² of tumor area), TLS area fraction (percentage of total tissue occupied by TLS), and binary presence/absence scoring (8, 11, 12). Gene expression signatures offer an alternative or complementary approach, with validated panels including 12-chemokine signatures (*CXCL13*, *CCL19*, *CCL21*), 7-gene signatures (*CXCL13*, *CCL19*, *MS4A1*, *LTB*, *CD37*, *CORO1A*, *IKZF1*), and broader 17–23 gene panels incorporating B-cell, T-cell, and dendritic cell markers (13–15). These transcriptomic approaches enable TLS assessment in archival samples lacking tissue for IHC but may not capture spatial organization or maturation status as precisely as histological methods. The lack of standardization across these methodologies significantly impacts cross-study comparisons and limits clinical implementation, highlighting the need for consensus guidelines on TLS assessment in genitourinary malignancies (9, 14).

### 2.5 Other classification systems

Some studies employ a morphology-based classification distinguishing early TLS (E-TLS), primary follicle-like TLS (PFL-TLS), and secondary follicle-like TLS (SFL-TLS) (7, 16–18). Secondary follicle-like TLSs resemble mature GC-like TLS with visible GCs on H&E staining or CD23+ follicular dendritic cells. This classification focuses on the presence of GCs as the critical distinguishing feature of mature TLS.

### 2.6 Clinical significance of maturation stages

The maturation stage directly correlates with functional capacity and clinical outcomes. Mature GC-like TLS with functional GCs demonstrate superior prognostic value compared to immature lymphoid aggregates, particularly in predicting immunotherapy response (19). This reflects the enhanced capacity of mature TLS to support local adaptive immune responses, including B-cell affinity maturation, plasma cell differentiation, and generation of tumor-reactive antibodies (20, 21).

## 3 TLS in prostate cancer

Prostate cancer (PCa) usually has an immunologically inactive TME with few lymphocytic infiltrations and rare TLS development (22). However, cases of spontaneous tumor regression have been reported to display mature TLSs with active GCs along with CD4+ and CD8+ T cell infiltration (23). These regions have also been found to be COX-2 negative and have fewer numbers of regulatory T-cells, suggesting that local immunosuppression modulate TLSs density (23). Targeting pathways like prostaglandin or stromal signaling should enhance TLS induction and increase response to immunotherapy (23). Although there are no trials currently focusing on TLSs in PCa, blockade of stroma and prostaglandin-mediated suppression may potentially help to facilitate TLSs induction and responses to immunotherapy.

### 3.1 Localized prostate cancer

In PCa, the presence of TLSs has gained interest due to their role in immunomodulation, therapy, and prognosis (24, 25). In localized PCa, TLSs can be found in peri-tumoral or intra-tumoral regions (24). These structures are enriched in immune-active gene expression, containing T-cell and B-cell related clusters and MHC class I/II (24). The presence of TLSs is also negatively associated with immunosuppressive profiles, such as T-regulatory cells and myeloid-derived suppressor cells; suggesting a role in counteracting immune evasion (24).

TLS formation in PCa may be driven by chemokines and cytokines such as chemokine (C-X-C motif) ligand (CXCL)-13 and interleukin (IL)-7, which promote lymphoid cells recruitment and the formation of HEVs (1). Additionally, TLS maturation and immunologic support are regulated through lymphotoxin-b receptor signaling, which coordinates immune-stromal communication and chemokine production (26). Classically, PCa’s are classified as immunologically “cold”, but TLS-positive tumors can have CD8+ T-cell infiltration, which is related to improved outcomes and reduced recurrence (27). This antitumor potential is enhanced in mature TLSs that contain follicular dendritic cells and GCs, by supporting antigen presentation and B-cell activation (6).

The presence of TLSs is linked to greater CD8+ T-cell infiltration, higher Th1 responses, and reduced Treg density, which work to slow tumor growth and increased immune-mediated cytotoxicity (5, 27–29). However, in some cases, poorly organized or immature TLSs may fail to create an appropriate immune response and instead be a niche for suppressive immune cell clusters (24, 30). Thus, TLSs maturity appears to play a role in determining their effects on disease progression. The physical location of TLSs within PCa’s also plays a role in modulating the TME. Peri-tumoral TLSs are related to antigen presentation pathways, while intra-tumoral TLSs are more associated with T-cell-related activity (24). Generally, TLSs have been associated with better prognosis across many malignancy types, and preliminary data suggest similar implications in PCa, opening the door for the development of new prognostic and therapeutic modalities (6).

### 3.2 Metastatic prostate cancer

TLSs are less frequently found in metastatic PCa, especially in bone and liver metastases (30). The sites of metastasis are dominated by immunosuppressive cytokines and are characterized by the absence of TLSs and reduced antigen expression, contributing to immune escape and resistance to treatment (30, 31).

The absence or dysfunction of TLSs in metastatic disease contributes to the poor response rates observed with immune checkpoint inhibitors in PCa. For example, trials using PD-1 and CTLA-4 blockade have shown modest results, possibly due to a lack of organized immune zones within the tumor or low T-cell infiltration (31). Therefore, induction of TLSs formation through cytokine modulation or LTbR activation may improve immune recognition and decrease resistance to immunotherapy (1, 6, 26, 27).

Another promising use of TLSs lies in the heterogeneity of these structures in PCa, which highlights the need for TLS-based stratification. Patients with high TLSs density and mature structures may benefit from immunotherapy, whereas those lacking them might require TLSs-priming strategies before checkpoint inhibition. As a result, TLS profiling could be used as a guide to optimize treatment strategies (6, 24). Of note, while most of the existing data highlight their protective role, certain subsets of these TLSs may facilitate tumor survival (27, 30). Immature TLSs or those with immunosuppressive cellular environments via infiltration of T-regs and M2 macrophages, which reduce the efficacy of checkpoint blockade and other immune-based therapies, may promote disease progression (27, 30, 31). Therefore, it is essential to understand the cellular composition and maturation stage of TLSs. To achieve this, research has proposed developing a staging system for TLSs from early aggregates to structures with fully functional GC, which may help predict outcomes more accurately (1, 6).

Looking forward, a multi-modal approach combining TLS induction, immune checkpoint inhibition, and androgen deprivation therapy may offer synergistic benefits in the treatment of PCa’s. This would address both the immunosuppressive TMEs and the hormonal dependence of PCa’s, while promoting immune infiltration and memory T-cell generation (26, 31).

## 4 TLS in bladder cancer

Existing data regarding TLSs in malignancies of the urinary bladder also agree on their potential as prognostic biomarkers and targets for immunotherapy. TLSs have been identified in both non-muscle invasive bladder cancer (NMIBC) and muscle-invasive bladder cancer (MIBC), though their frequency and maturation differ significantly by tumor grade and stage (32). TLSs were more frequently observed in high-grade NMIBC and MIBC than in low-grade NMIBC, suggesting that TLS presence increases with tumor stage and grade (32). This TLS enrichment in advanced disease reflects the establishment of immune-inflamed tumor microenvironment (TME) characterized by enhanced infiltration of CD8+ T cells, B cells, and mature dendritic cells, along with upregulation of effector molecules such as GZMA, IFNG, and PDCD1 (15, 33). Importantly, the prognostic benefit of TLS is treatment-dependent; while TLS density and maturation predict superior responses to BCG therapy in NMIBC and immune checkpoint inhibitors in MIBC, they show no association with outcomes following neoadjuvant chemotherapy alone (32, 34, 35). In this context, mature TLS with GC formation predict BCG and ICI responsiveness, potentially offsetting the adverse biology associated with higher tumor stage and grade through coordinated local adaptive immune responses.

### 4.1 TLS in early-stage non-muscle invasive bladder cancer

While TLSs are present in early-stage bladder cancer, they tend to be less mature and organized in NMIBC, potentially due to less chronic inflammation in early stages of the disease (1, 36). Spatial transcriptomic analysis has provided further insight, identifying the colocalization of CXCL13^+^ CD4^+^ T-cells and CXCR^+^ NR4A2^+^ B-cells within TLSs regions, highlighting the CXCL13-CXCR5 axis as a key mechanism in TLS formation (15). Mature TLS, marked by structured, well-defined, B-cell zones and follicular dendritic cell networks, were more commonly observed in MIBC tumors, underscoring the progressive maturation in TLSs in the setting of advancing disease (15).

The presence and density of TLSs in NMIBC cancer has demonstrated a strong association with improved response to Bacillus Calmette-Guerin (BCG) therapy. In a cohort analysis, 62.5% of patients with high TLS density achieved a complete response to BCG, compared to only 27.3% in the low TLS density group, further cementing the value TLSs have in achieving favorable therapeutic responses (32). Additionally, NMIBC patients whose tumors had a high TLSs signature scores experienced significantly longer recurrence-free survival (RFS) and had greater infiltration of CD8^+^ T cells and B cells, both critical for anti-tumor immunity (36). These findings may be attributed to TLSs enhancing BCG efficacy by enabling a coordinated immune response through the recruitment and activation of B-cells, dendritic cells, and T cells within the TME (1).

TLSs have also emerged as promising prognostic biomarkers in NMIBC. Although TLSs were less frequently observed in NMIBC compared to muscle-invasive disease, their presence still correlated with improved survival outcomes, including in BCG-treated patients, though this did not reach statistical significance in some analyses (15). Regardless, TLS signature scores were independently associated with increased RFS and progression-free survival (PFS) in NMIBC patients and high TLS scores were linked to better disease-free survival (DFS) across three independent bladder cancer cohorts (37, 38). Lastly, retrospective analyses also identified TLSs as a biomarker of reduced recurrence and progression risk in NMIBC (1).

### 4.2 TLS in locally advanced bladder cancer

In MIBC, TLSs are more prevalent and structurally mature compared to those in early-stage disease; supporting the idea of increased density and organization with advanced tumor biology (39). At the immunological level, TLSs were positively correlated with CD8^+^ T cells, dendritic cells, and M1 macrophages, and negatively associated with immunosuppressive M2 macrophages and T-regs, supporting the evidence that mature, functioning TLSs contribute to a tumor-suppressive microenvironment (40). In a study by Ligon et. al., TLSs were observed in 60% of MIBC tumors, predominantly as mature follicular TLS (FL-TLS) characterized by CD21^+^ follicular dendritic cells and GC-like architecture (41). Additionally, a high TLS signature score in patients receiving anti-PD-1/PD-L1 therapy resulted in improved objective response rate (ORR) and (PFS), reinforcing the evidence of the significant role these TLSs play therapy response (36).

TLSs in MIBC are strongly associated with an immune-inflamed TME characterized by enhanced immune cell infiltration and functional activation. Correspondingly, gene expressions within TLS-enriched zones showed elevated levels of effector molecules such as GZMA, IFNG, and PDCD1, and signatures of tumor-reactive T-cells were significantly increased in TLS-positive MIBC samples (15). Plasma cell numbers were also increased in TLS-positive environments, and secreted immunoglobulin heavy constant gamma (IGHG)-1 and IGHG3 which are immunoglobulin isotypes associated with antibody-dependent cellular phagocytosis (15).

In MIBC, TLS-rich tumors also exhibit increased densities of CD8^+^ T cells, CD20^+^ B cells, and DC-LAMP^+^ dendritic cells, suggesting a robust immune landscape and a highly inflamed immune tumor milieu (15, 37). Immune cell densities were greatest within TLSs regions of interest and decreased with distance from TLSs, emphasizing their localized immunological activity (42). Furthermore, follicular TLSs (FL-TLSs) contained higher B-cell densities and occupied larger spatial domains than early TLSs (E-TLSs), further supporting the relation between structural maturity and adequate function (42). These structurally mature TLSs seen in MIBC, are also associated with elevated CD8^+^ T-cell infiltration, which further highlights the immune-inflamed tumor phenotype (1). Together, these organized structures promote antigen presentation via follicular dendritic cells and mature dendritic cells and coordinate T and B-cell zones, thereby establishing a localized immune-responsive niche within the TME (39).

The presence of TLSs and their associated genetic signatures have been linked to favorable responses to immune checkpoint blockade in MIBC. In the IMvigor210 cohort, patients with higher TLSs gene signature scores exhibited significantly improved overall survival (OS) following anti-PD-L1 therapy (15). A 7-gene TLS-specific signature, comprising CXCL13, CCL19, MS4A1, LTB, CD37, CORO1A, and IKZF1, was developed to stratify patients by their response to checkpoint inhibitors (15). Similarly, TLS score was a strong predictor of complete or partial response among anti-PD-L1-treated patients in IMvigor210 (38). Notably, patients categorized as TLS-high-PMN-MDSC-low demonstrated the best prognoses, while TLS-low-PMN-MDSC-high had the poorest outcomes (42). TLS signatures, including 12-chemokine and CXCL13-based models, were also associated with enhanced survival in the IMvigor210 trial (42). Multiple large-scale studies, such as the IMvigor210 cohorts, have confirmed elevated TLS gene expression in responders to PD-1/PD-L1 blockade (1). Clinical evidence continues to demonstrate that TLS-rich tumors in bladder cancer respond more favorably to checkpoint blockade therapy, and high TLS signature scores are also associated with elevated expression of immune checkpoint molecules and superior anti-PD-L1 therapeutic response (39, 40).

TLS structures also appear to positively influence therapeutic responsiveness within the context of neoadjuvant therapies. In the BLASST-1 trial, which evaluated neoadjuvant nivolumab, gemcitabine, and cisplatin followed by radical cystectomy in patients with MIBC, transcriptome-wide profiling of 37 pretreatment transurethral resection of bladder tumor (TURBT) specimens was performed to identify signatures predictive of pathological response rate (PaR) and pathological complete response (PCR) (43). Higher immune activation signatures (which serve as a proxy for TLS presence) and other immune-rich signatures correlate with better PaR in the TME (43). These findings further support the role of TLS in establishing an immune-inflamed TME that enhances therapeutic sensitivity and contributes to improved treatment outcomes.

### 4.3 TLS in metastatic bladder cancer

TLSs are found in both primary and metastatic bladder cancer, though those in metastases are typically less mature and organized (44). To better characterize TLS in this context, a seven-gene TLS-related signature, comprising *CXCL13*, *CCL19*, *CD79A*, *CD27*, *MS4A1*, *LTB*, and *SELL*, was developed using LASSO regression to predict TLS presence and immune activity across primary and metastatic disease (38).

TLSs play a key role in systemic immune responses in bladder cancer. A conserved immune profile, including TLS-related gene signatures, is seen across matched primary and metastatic tumors, suggesting TLSs contribute to a unified systemic immune phenotype (44). In metastatic blader cancer, TLS score was also found to positively correlate with increased CD8^+^ T cell infiltration, dendritic cell abundance, and M1 macrophages, B-cell receptor signaling genes, class-switched immunoglobulins, and antigen presentation components, while showing a negative correlation with M2 macrophages and Tregs, indicating a tumor-suppressive immune landscape (38, 41).

TLS-associated gene signatures, particularly those including *CXCL13* and *CCL19*, have also been validated in multiple cohorts treated with PD-1/PD-L1 blockade, further underscoring their role in mediating antitumor immunity at both local and systemic levels (33). Patients with high TLS gene expression signatures in metastatic lesions also exhibited similar r benefits resulting in longer PFS following checkpoint blockade therapy (44). Integration of TLSs mapping with digital pathology techniques enables spatial identification of immune-enriched regions that correlate with treatment response and survival outcomes (36). Among patients treated with anti-PD-1 or PD-L1 agents, those with high TLS scores demonstrated higher ORR (42% vs. 19%) and longer OS (median not reached vs. 9.4 months) (37).

Given these findings, an immune scoring approach based on TLS high PMN-MDSC low has been proposed to help identify patients most likely to benefit from immune checkpoint blockade (42). Additionally, in support of translational strategies, induction of TLSs through cytokines such as LIGHT and CCL21 or via dendritic cell vaccines has shown preclinical efficacy in enhancing antitumor immunity (33).

Bladder cancer has been a major focus of research investigating how immune checkpoint therapies may drive TLS development. One example is the phase 1b NABUCCO trial, which evaluated neoadjuvant ipilimumab and nivolumab in patients with MIBC. The combination targets two key immune checkpoints, CTLA-4 and PD-1. During the study, 24 patients were treated with two doses of ipilimumab followed by nivolumab. It was found that 46% of participants showed no viable tumor at the time of cystectomy post-treatment (45). Upon histological and molecular analysis, a subset of specimens demonstrated apparent TLS formation, which was defined by dense lymphocytic aggregates, the presence of follicular dendritic cells, and the evidence of B-cell activation and immune infiltration (45). The finding supported that the checkpoint blockade may not only enable the formation of TSLs within tumors but also elicit antitumor immunity (45). Within the context of dual checkpoint blockade, another study analyzed the effects of durvalumab/tremelimumab combination in cisplatin-ineligible patients. The study showed promising results with a viable tumor rate of 37.5% and a downstaging rate of 58% (46). While TLSs were not directly quantified, upregulation of B-cell associated genes was observed in responding tumors using transcriptomic analysis, consistent with TLS activity (46).

### 4.4 Ongoing trials

Currently, ongoing trials aim to better understand and enhance TLS responses. The SURE-01 trial evaluates pembrolizumab, a PD-1 inhibitor, as neoadjuvant therapy in cisplatin-ineligible patients, incorporating tissue analysis to assess changes in TLS density and maturity (47). The NURE-Combo trial studies nivolumab with nab-paclitaxel to determine whether this combination induces immune infiltration and promotes TLS formation (48, 49). The SURE-02 trial tests pembrolizumab with sacituzumab govitecan, targeting Trop-2, to evaluate the effects of combining immune checkpoint inhibition with targeted cytotoxic therapy on TLS induction and maintenance (50).

## 5 TLSs in testicular cancer

TLSs in the setting of testicular cancer is an area of emerging interest, and data describing their role and significance in these tumors are limited. Generally, the testes are an immune-privileged site, but inflammation can disrupt this environment and promote immune-cell infiltration. In the setting of testicular malignancies, TLSs contribute to the local immune response and may influence tumor progression and spread. Their presence is often associated with stronger immune activity and better prognosis, while their absence or suppression may reflect a more aggressive pathology.

### 5.1 Localized testicular cancer

In a model of autoimmune orchitis, TLSs resembling secondary lymphoid structures consisting of B-cells, T-cells, follicular dendritic cells, and HEVs have been observed; indicating local antigen processing and adaptive immune activity (51). In these models, TLS formation in the testes was associated with exposure to spermatic antigens and characterized by upregulation of the lymphoid-organizing chemokines CXCL13 and CCL19 (51). Their formation was observed in areas adjacent to seminiferous tubule destruction, suggesting an immune response to localized antigen release (51). The reported upregulation of chemokines such as CXCL13 and CCL19 in these settings further provides evidence of lymphocyte aggregation and TLS maturation (51). Although these findings have been studied in the setting of an infectious process, they provide an opportunity to understand the role of TLSs in the testes.

TLSs have sporadically been identified in testicular tumors, especially seminomas, and they are typically associated with areas of dense lymphoid infiltration (1). Current data suggest that TLSs positive tumors play a role in local anti-tumor immune responses. Notably, the proximity of TLSs to intratumoral vessels and necrotic areas in seminomas suggests that antigen release from tissue damage may play a role in their formation (1). These findings suggest active local immune responses with organized B-cell and T-cell areas and mature dendritic cells (1). Unlike autoimmune orchitis models-where TLS formation is linked to spermatic antigen exposure and chemokine upregulation-TLS-like structures in seminomas appear near dense lymphoid infiltrates, though the exact factors driving their formation remain unknown (1, 51).

In testicular germ cell tumors (TGCTs), differences in TLS composition and presence are evident between seminoma and non-seminoma tumor subtypes. Of note, the presence of TLSs in seminomas without evidence of their suppression in the TME - along with their slow progression and favorable prognosis underscores the possible role of TLSs in slowing tumor progression and spread (1, 52). In contrast, non-seminomatous germ cell tumors - particularly embryonal carcinoma (EC)-exhibit a distinct immune profile, with evidence of active TLS suppression (52). Single-cell RNA sequencing has shown that high SERPINB9 expression in metastatic EC correlates with downregulation of TLS-related chemokines (CXCL13, IL-6, IL-6, and CCL5), suggesting a reduced capacity to recruit and organize immune cells into TLS-like structures (53). This immune suppression may hinder local anti-tumor immune response. SERPINB9 expression is also associated with increased tumor stemness features and greater metastatic potential, suggesting a broader role in shaping an immunosuppressive TME (53). Collectively, these findings highlight fundamental differences in TLS presence and immune organization between seminomas and non-seminoma TGCTs and highlights the need for further research in the role and significance of TLSs across testicular malignancies.

### 5.2 TLSs in metastatic testicular germ cell tumors

In testicular cancer, metastasis often occurs via lymphatic spread to retroperitoneal nodes - and the environment may either support or inhibit TLS formation depending on the tumor’s immune-modulating capabilities (53). SERPINB9 expression in metastatic sites has been associated with suppression of TLS-promoting chemokines, which may contribute to an immune-suppressive environment and resistance to immune checkpoint therapy (53). Their absence, especially in tumors expressing immunosuppressive mediators like SERPINB9, is associated with more aggressive tumor behavior and greater incidence of therapeutic resistance (53). This association serves as a promising area of study for predicting and monitoring tumor behavior and for developing new therapies.

TLS status could serve as a biomarker in testicular cancer management and immunotherapy planning. Moreover, modulating TLS-inducing chemokines and targeting suppressors genes may provide a foundation for TLS-focused immunotherapies. Further, insights from other malignancies suggest that strategies aimed at promoting TLS formation may hold promise in inducing immune responses in tumors with poor baseline immunogenicity (1, 52).

## 6 TLS in kidney tumors

In clear cell renal cell carcinoma (ccRCC), TLSs are relatively common; however, their prognostic value remains context dependent. Much of the available evidence links the presence of TLSs to improved clinical outcomes; but current data also associate specific TLS phenotypes, maturity levels, or chemokine signatures - particularly elevated CXCL13 - with more aggressive disease and poorer prognosis (14).

Compared to ccRCC, TLSs in papillary RCC (pRCC) and chromophobe RCC (chRCC) are infrequently observed (54). The immune TME in these subtypes is typically less inflamed, characterized by reduced densities of tumor-infiltrating lymphocytes (TILs) and TLS. Such differences potentially explain their diminished responsiveness to immunotherapy and a limited prognostic impact associated with TLS presence (54–56).

### 6.1 TLS in early-stage kidney tumors

TLSs identified in early-stage renal cell carcinoma (RCC) correlates with enhanced infiltration of immune effector cells, particularly CD8^+^ cytotoxic T-cells and B-cells, promoting an inflammatory TME that may support both cellular and humoral antitumor immunity (54). Additionally, specific chemokines, most notably CXCL13, CCL19, and CCL21, have been identified as essential mediators in TLS formation, organization, and function. In ccRCC with high TLSs density, it was found that expression of these chemokines was significantly higher compared to TLSs negative tumors (54). The increase chemokine expression was also observed to correlate with higher immune effector cell infiltration (54). Interestingly, although TLSs are associated with a better prognosis, expression of CXCL13 was specifically associated with shorter progression-free and OS (14, 54).

In ccRCC, the presence of mature TLSs containing well-developed GCs is associated with significantly improved clinical outcomes (54). These patients with TLS-rich tumors experienced longer OS and progression-free survival (54). TLS-rich tumors were also associated with higher expression of immune checkpoint–relevant markers and greater predicted response scores to PD-1 and CTLA-4 inhibitors (54). These findings highlight the prognostic and therapeutic value of structurally mature and functional TLSs in ccRCC.

### 6.2 TLS in metastatic kidney tumors

In their study, 57, analyzed matched primary tumors (PTs) and distant metastases (METs) from 47 patients with ccRCC. Digital spatial profiling was used to assess CD8^+^ Tcell distribution, co-expression of the exhaustion marker TOX, and to quantify TLS density (57). Most METs displayed an immune “cold” phenotype, defined by low densities of lymphocytes and TLSs (57). However, a small subset of METs classified as “inflamed” exhibited larger cumulative TLS areas and higher proportions of CD8^+^TOX^+^ T-cells compared to their matched PTs subset (57). At the same time, they observed that “hot” PTs, with dense CD8^+^ infiltration and increased CD8^+^TOX^+^ T-cells, were associated with shorter disease-free survival, suggesting that high immune cell presence may reflect T-cell exhaustion rather than effective immune control (57). These findings support the idea that immune features, including high TLSs density, may progress with advancing disease and evolve into dysfunctional units (57). In this context, TLSs may be used as highly informative biomarkers for stratifying disease progression, staging, and treatment options.

## 7 TLS in Penile Cancer

The TME of penile cancer has become an area of growing interest, particularly with respect to TLSs. These ectopic lymphoid aggregates are increasingly recognized as important modulators of local immunity in solid tumors, but their significance in penile malignancies is poorly understood.

### 7.1 TLS in localized penile cancer

In localized penile squamous cell carcinoma (SCC), TLSs have been identified within the tumor parenchyma and surrounding stromal regions, indicating active local immune engagement (58). As seen in many other TLSs positive solid tumors, IHC analyses revealed that TLS-positive penile SCC exhibited higher infiltration of CD8+ T-lymphocytes and CD20+ B cells, along with more mature dendritic cells, compared to TLS-negative tumors (58). Of significant interest however, it was found that TLS positive penile SCCs were independently associated with better OS, regardless of tumor stage or nodal status, suggesting that localized immune responses within the TME significantly influences disease progression (58). Additionally, TLS-positive tumors exhibited upregulation of genes related to immune activation, including markers of cytotoxic T-cell activity such as granzyme B (GZMB) and interferon gamma (IFNG) (58). These tumors also had lower expression of immunosuppressive cytokines compared to their TLS-negative counterparts, suggesting a favorable environment for anti-tumor immune responses (58). These findings have been substantiated by a robust multicenter cohort of 165 penile SCC cases, in which patients with fewer TLSs demonstrated significantly worse OS with hazard ratio (HR) of 2.17 (95% CI: 0.94-5; *P* = 0.069). High B-cell immunoscore (reflecting CD20+ and CD138+ cell densities within and around TLS) was independently associated with improved OS with HR of 1.89 (95% CI: 1.18-3.03; *P* = 0.008) (59). Notably, high TLS diameter correlated with brisk lymphocytic infiltrate (OR = 2.24; 95% CI: 1.10-4.55; *P* = 0.021), and high B-cell immunoscores were strongly associated with mutated *p53* profiles and enhanced immune activation (59, 60). Recent spatial transcriptomic analyses have further revealed that CD74+ B cells within TLS are enriched during early TLS formation and engage with naïve T cells through HLA-DRA-mediated interactions, activating key transcription factors (*NFKB1*, *NFKB2*, *NFATC1*, *FOS*, *RUNX1*) that enhance local immune responses and correlate with improved patient survival (61).

Further supporting this data, TLS-positive tumors were found to have lower serum levels of squamous cell carcinoma antigen (SCCAg), a biomarker that correlates to increased tumor burden and poor prognosis (62). These observations hint at the prominent role these structures have on penile SCC progression, and to their value as prognostic and therapeutic targets.

Lymphovascular invasion is a well-established adverse prognostic factor in penile SCC, and the presence of robust local immune responses may theoretically help limit distant spread (63). Supporting this statement, the study by Yi et al. (64) showed that weak local immune control at the primary tumor site in penile SCC is linked to early lymphatic spread and pelvic node metastasis (64). Although primary data are limited, in the context of the general findings by Yi et al. (64) and the established data supporting TLSs role in modulating antitumor immunity in solid tumors, it is worth noting the crucial role that TLSs may have in preventing penile SCC metastatic spread (64).

Beyond TLS-specific investigations, recent immuno-oncology studies have characterized the broader tumor immune microenvironment in penile SCC (60, 65, 66), revealing critical insights into immune checkpoint expression, tumor-immune interactions, and mechanisms of immune evasion that provide biological context for TLS-mediated immune control (67). Inflammation plays a crucial role in penile cancer development and progression, with the tumor immune microenvironment characterized by tumor-associated macrophages, cancer-associated fibroblasts, and tumor-infiltrating lymphocytes that produce pro-inflammatory cytokines and chemokines associated with tumor progression (65). The nuclear factor kappa B (NF-κB) pathway and secreted phosphoprotein 1 (SPP1) have been implicated in penile cancer pathogenesis, while elevated C-reactive protein (CRP) levels and neutrophil-to-lymphocyte ratio (NLR) have been identified as potential prognostic biomarkers, with high NLR (≥3.0) associated with advanced stage, lymphovascular invasion, and immunosuppressive tumor microenvironment characterized by increased N2 tumor-associated neutrophils and CD8+ T-cell exhaustion (65, 68).

In a large retrospective study of 152 penile SCC cases, brisk lymphocytic infiltrate was an independent predictor of improved overall survival and cancer-specific survival (HR for non-brisk/absent infiltrate: 2.22; *P* = 0.0023) (69). Multiplex immunofluorescence studies have revealed progressive T-cell exhaustion with advancing disease stage. An initial immune response in early locoregional disease (N1) with increased CD3+, CD4+, and CD8+ T-cell densities is followed by immune exhaustion in advanced disease (N2-3), marked by declining cytotoxic T-cell density, rising PD-L1 expression, and progressive replacement of anti-tumor M1 macrophages with pro-tumorigenic M2 macrophages (70). Additional studies demonstrate that PD-L1 expression at the invasive margin is significantly elevated in node-positive disease, TILs exhibit higher PD-1 expression compared to peripheral blood, and immune checkpoint molecules (PD-1, LAG3, TIM3) are co-expressed in high-grade tumors (66, 69, 71).

Furthermore, spatial analysis demonstrates that close clustering of M2 macrophages with tumor cells is associated with worse OS, recurrence-free survival, and cancer-specific survival, whereas bivariate clustering of CD3+CD4+ helper T cells with tumor cells correlates with improved outcomes, including in node-positive disease (70). These findings support the biological plausibility of TLS-mediated immune control, where TLS presence may counteract the immunosuppressive mechanisms (including T-cell exhaustion, M2 macrophage predominance, and checkpoint molecule upregulation) that characterize advanced penile cancer, thereby reinforcing the rationale for both TLS assessment as a prognostic biomarker and immune checkpoint inhibitor-based therapeutic strategies in this disease (72–75).

### 7.2 TLS in metastatic penile cancer

In metastatic penile SCC, TLSs have been identified within both inguinal and pelvic lymph nodes (58). IHC analyses demonstrated that metastatic lymph nodes containing TLSs exhibited higher densities of CD8+ T cells and CD20+ B cells relative to TLS-negative nodes, suggesting that TLS presence also sustain active local immune surveillance in metastatic lesions (58).

In penile squamous cell carcinoma (SCC), TLSs were observed more frequently in primary tumors from patients who did not have distant metastases, compared to those with metastatic disease (58). This pattern hints at an inverse relationship between TLS presence and the presence of metastatic lesions; though the concrete reason for this association remains unknown (58).

Overall, although current data are limited, these findings collectively suggest that TLS evaluation in penile SCC could provide valuable prognostic and therapeutic information for the management of these malignancies.

## 8 Emerging artificial intelligence-based technology and future directions

Artificial intelligence (AI)-based computational pathology methods for automated TLS detection on H&E slides represent a practical pathway toward standardization, with deep learning models achieving high Dice coefficients (up to 0.866-0.91) for TLS segmentation and demonstrating strong prognostic value across different cancer types (76). These AI approaches can be integrated into the clinical workflows through standardized protocols that combine H&E-based automated detection with CD23 IHC validation, enabling reproducible quantification of TLS density, maturation status, and spatial distribution (9).

For bladder cancer and RCC specifically, AI-based spatial analysis of TILs on H&E slides has shown significant correlation with immunotherapy outcomes (77). In a cohort of 56 metastatic urothelial carcinoma (UC) patients treated with immune checkpoint inhibitors, computational features related to spatial architectural patterns of TILs predicted OS with HR of 1.90 (95% CI 0.97-3.73, *p* = 0.036) (78). The UC-TIL classifier, which quantifies spatial TIL patterns from H&E slides, achieved AUC = 0.757 for predicting immunotherapy response and identified non-responders with 91% specificity in metastatic disease (77).

In RCC, TLS presence correlated with IgG-producing plasma cells and tumor cell apoptosis, with therapeutic responses to immune checkpoint inhibitors correlating with IgG-stained tumor cells (21, 79). B cell signatures and TLSs were the most differentially expressed features between responders and non-responders to immune checkpoint inhibitors in melanoma and RCC cohorts, establishing the biological rationale for AI models to prioritize TLS and B-cell features (79).

A practical standardization framework for GU cancers should include: (1) Primary screening with automated H&E-based TLS detection using validated deep learning models with defined performance thresholds (76); (2) Maturation assessment (stages 1, 2, and3) based on architectural features (9, 80); (3) Validation protocol such as CD23 IHC on cases with borderline or uncertain maturation status (9); (4) Standardized quantification metrics for reporting TLS density (per mm²), TLS ratio (area percentage), and maturation distribution (12, 76); (5) and Quality control. In addition, in order to enhance immunotherapy response prediction in GU cancer especially RCC and bladder cancers, multimodal frameworks should incorporate TLS features alongside established biomarkers, including B-cell markers (21, 79), chemokine signatures (81, 82), and spatial TIL patterns (3).

Integration of these features with clinical variables (TNM staging) has been shown to significantly enhance discriminative ability for OS prediction in 10 out of 15 TCGA tumor types (76). For bladder cancer specifically, TLS pattern-based scoring systems that incorporate gene expression signatures of 39 validated TLS signature genes have demonstrated superior prediction of immunotherapy response compared to tumor mutational burden alone (39).

## 9 Conclusions

Despite increasing interest in TLSs in the setting of GU malignancies, their functional role remains incompletely understood. The heterogeneity of their role and variable disease-modifying significance across different types of malignancies highlights the need for further research on the topic of TLSs. Standardized criteria for evaluating TLSs in both primary and metastatic lesions are needed to clarify their prognostic and therapeutic relevance. Grossly, the existing evidence links TLS presence to stronger immune activation, better survival, and greater response to systemic therapies, suggesting that integrating TLS-based patient stratification to current practices could enhance prognostic accuracy and guide immunotherapy.

Therapeutic strategies such as chemokine modulation, dendritic cell activation, and lymphotoxin-β receptor targeting may promote TLS formation and function, boosting antitumor immunity. In GU malignancies, TLSs are increasingly recognized not just as biomarkers but as actionable targets, with their presence often correlating with improved outcomes. Advancing our ability to induce and sustain TLSs could support the development of more effective, personalized cancer therapies.

## References

[1] Shen C Zhang D-L Cheng X-L Zhang W-C Zhao J-J . Urological tumor: A narrative review of tertiary lymphatic structures. Urol Int. (2023) 107:841–7. doi: 10.1159/000532127, PMID: 37769625 PMC10623398

[2] Schumacher TN Thommen DS . Tertiary lymphoid structures in cancer. Science. (2022) 375:eabf9419. doi: 10.1126/science.abf9419, PMID: 34990248

[3] Van Dijk N Gil-Jimenez A Silina K Van Montfoort ML Einerhand S Jonkman L . The tumor immune landscape and architecture of tertiary lymphoid structures in urothelial cancer. Front Immunol. (2021) 12:793964. doi: 10.3389/fimmu.2021.793964, PMID: 34987518 PMC8721669

[4] Yu A Fan Z Ma L Tang J Liu W Han Z . The relationship between the tertiary lymphoid structure and immune-infiltrating cells in gastrointestinal cancers: A systematic review and meta-analysis. Immunity Inflammation Dis. (2024) 12:e70003. doi: 10.1002/iid3.70003, PMID: 39259184 PMC11389262

[5] Koti M Bivalacqua T Black PC Cathomen T Galsky MD Gulley JL . Adaptive immunity in genitourinary cancers. Eur Urol Oncol. (2023) 6:263–72. doi: 10.1016/j.euo.2023.03.002, PMID: 37069029

[6] Dieu-Nosjean M-C Goc J Giraldo NA Sautès-Fridman C Fridman WH . Tertiary lymphoid structures in cancer and beyond. Trends Immunol. (2014) 35:571–80. doi: 10.1016/j.it.2014.09.006, PMID: 25443495

[7] Le Rochais M Hémon P Ben-Guigui D Garaud S Le Dantec C Pers JO . Deciphering the maturation of tertiary lymphoid structures in cancer and inflammatory diseases of the digestive tract using imaging mass cytometry. Front Immunol. (2023) 14:1147480. doi: 10.3389/fimmu.2023.1147480, PMID: 37143660 PMC10151544

[8] Barmpoutis P Di Capite M Kayhanian H Waddingham W Alexander DC Jansen M . Tertiary lymphoid structures (TLS) identification and density assessment on H&E-stained digital slides of lung cancer. PloS One. (2021) 16:e0256907. doi: 10.1371/journal.pone.0256907, PMID: 34555057 PMC8460026

[9] Vanhersecke L Bougouin A Crombé A Brunet M Sofeu C Parrens M . Standardized pathology screening of mature tertiary lymphoid structures in cancers. Lab Invest. (2023) 103:100063. doi: 10.1016/j.labinv.2023.100063, PMID: 36801637

[10] Klein C Devi-Marulkar P Dieu-Nosjean MC Germain C . Development of tools for the selective visualization and quantification of TLS-immune cells on tissue sections. Methods Mol Biol. (2018) 1845:47–69. doi: 10.1007/978-1-4939-8709-2_4, PMID: 30141007

[11] Kushnarev V Belozerova A Dymov D Popov Y Lukashevich N Valiev I . A digital imaging analysis (DIA) platform for identifying tertiary lymphoid structures (TLS) in lung adenocarcinoma (LUAD). J Clin Oncol. (2022) 40:3142–2. doi: 10.1200/JCO.2022.40.16_suppl.3142

[12] Li Z Jiang Y Li B Han Z Shen J Xia Y . Development and validation of a machine learning model for detection and classification of tertiary lymphoid structures in gastrointestinal cancers. JAMA Network Open. (2023) 6:e2252553–e2252553. doi: 10.1001/jamanetworkopen.2022.52553, PMID: 36692877 PMC10408275

[13] Zhu G Falahat R Wang K Mailloux A Artzi N Mulé JJ . Tumor-associated tertiary lymphoid structures: gene-expression profiling and their bioengineering. Front Immunol. (2017) 8:767. doi: 10.3389/fimmu.2017.00767, PMID: 28713385 PMC5491937

[14] Cui X Gu X Li D Wu P Sun N Zhang C . Tertiary lymphoid structures as a biomarker in immunotherapy and beyond: Advancing towards clinical application. Cancer Lett. (2025) 613:217491. doi: 10.1016/j.canlet.2025.217491, PMID: 39862919

[15] Lin J Jiang S Chen B Du Y Qin C Song Y . Tertiary lymphoid structures are linked to enhanced antitumor immunity and better prognosis in muscle-invasive bladder cancer. Adv Sci (Weinh). (2025) 12:e2410998. doi: 10.1002/advs.202410998, PMID: 39739621 PMC11831474

[16] Siliņa K Soltermann A Attar FM Casanova R Uckeley ZM Thut H . Germinal centers determine the prognostic relevance of tertiary lymphoid structures and are impaired by corticosteroids in lung squamous cell carcinoma. Cancer Res. (2018) 78:1308–20. doi: 10.1158/0008-5472.CAN-17-1987, PMID: 29279354

[17] Liang H Zhang Z Guan Z Zheng S Lou J Liu W . Follicle-like tertiary lymphoid structures: A potential biomarker for prognosis and immunotherapy response in patients with laryngeal squamous cell carcinoma. Front Immunol. (2023) 14:1096220. doi: 10.3389/fimmu.2023.1096220, PMID: 36776859 PMC9912937

[18] Xie M Lin X Bao X Liang Y Deng H Song J . Tertiary lymphoid structure in tumor microenvironment and immunotherapy of lung cancer. Archivos Bronconeumología. (2024) 60:S77–85. doi: 10.1016/j.arbres.2024.07.020, PMID: 39174437

[19] Chen Y Wu Y Yan G Zhang G . Tertiary lymphoid structures in cancer: maturation and induction. Front Immunol. (2024) 15. doi: 10.3389/fimmu.2024.1369626, PMID: 38690273 PMC11058640

[20] Fridman WH Meylan M Petitprez F Sun CM Italiano A Sautès-Fridman C . B cells and tertiary lymphoid structures as determinants of tumour immune contexture and clinical outcome. Nat Rev Clin Oncol. (2022) 19:441–57. doi: 10.1038/s41571-022-00619-z, PMID: 35365796

[21] Meylan M Petitprez F Becht E Bougoüin A Pupier G Calvez A . Tertiary lymphoid structures generate and propagate anti-tumor antibody-producing plasma cells in renal cell cancer. Immunity. (2022) 55:527–41. doi: 10.1016/j.immuni.2022.02.001, PMID: 35231421

[22] Abou-Kheir W . Tumor microenvironment in prostate cancer: toward identification of novel molecular biomarkers for diagnosis, prognosis, and therapy development. Front Genet. (2021) 12. doi: 10.3389/fgene.2021.652747, PMID: 33841508 PMC8033163

[23] García-Hernández MDLL Uribe-Uribe NO Espinosa-González R Kast WM Khader SA Rangel-Moreno J . A unique cellular and molecular microenvironment is present in tertiary lymphoid organs of patients with spontaneous prostate cancer regression. Front Immunol. (2017) 8:563. doi: 10.3389/fimmu.2017.00563, PMID: 28567040 PMC5434117

[24] Shahait M Hakansson AK Daniel RE Hosny K Davicioni E Liu SY . Quantification and molecular correlates of tertiary lymphoid structures in primary prostate cancer. Prostate. (2024) 84:709–16. doi: 10.1002/pros.24684, PMID: 38544351

[25] Lara Hilal MS Mukherji D Charafeddine M Farhat Z Temraz S Khauli R . Prostate cancer in the arab world: A view from the inside. Clin Genitourin Cancer. (2015) 13:505–11. doi: 10.1016/j.clgc.2015.05.010, PMID: 26149392

[26] Novysedlak R Guney M Al Khouri M Bartolini R Koumbas Foley L Benesova I . The immune microenvironment in prostate cancer: A comprehensive review. Oncology. (2024) 103:521–45. doi: 10.1159/000541881, PMID: 39380471 PMC12140600

[27] Wolf MJ Seleznik GM Zeller N Heikenwalder M . The unexpected role of lymphotoxin β receptor signaling in carcinogenesis: from lymphoid tissue formation to liver and prostate cancer development. Oncogene. (2010) 29:5006–18. doi: 10.1038/onc.2010.260, PMID: 20603617

[28] Tsai YC Chen WY Abou-Kheir W Zeng T Yin JJ Bahmad H . Androgen deprivation therapy-induced epithelial-mesenchymal transition of prostate cancer through downregulating SPDEF and activating CCL2. Biochim Biophys Acta Mol Basis Dis. (2018) 1864:1717–27. doi: 10.1016/j.bbadis.2018.02.016, PMID: 29477409

[29] Gogola S Rejzer M Bahmad HF Abou-Kheir W Omarzai Y Poppiti R . Epithelial-to-mesenchymal transition-related markers in prostate cancer: from bench to bedside. Cancers (Basel). (2023) 15. doi: 10.3390/cancers15082309, PMID: 37190236 PMC10136789

[30] Li D Xu W Chang Y Xiao Y He Y Ren S . Advances in landscape and related therapeutic targets of the prostate tumor microenvironment. Acta Biochim Biophys Sin. (2023) 55:956–73. doi: 10.3724/abbs.2023092, PMID: 37294106 PMC10326416

[31] Jansen CS Prokhnevska N Kissick HT . The requirement for immune infiltration and organization in the tumor microenvironment for successful immunotherapy in prostate cancer. Urologic Oncol Semin Original Investigations. (2019) 37:543–55. doi: 10.1016/j.urolonc.2018.10.011, PMID: 30446449 PMC6513714

[32] Yilmaz F Sagir S . Prognostic and predictive value of tertiary lymphoid structures in TURBT materials: Should it be seated in the routine pathological examination, and can it be used in deciding on the treatment method? Urol Oncol. (2024) 42:450.e413–450.e422. doi: 10.1016/j.urolonc.2024.06.010, PMID: 39089974

[33] Pagliarulo F Cheng PF Brugger L Van Dijk N Van Den Heijden M Levesque MP . Molecular, immunological, and clinical features associated with lymphoid neogenesis in muscle invasive bladder cancer. Front Immunol. (2022) 12. doi: 10.3389/fimmu.2021.793992, PMID: 35145509 PMC8821902

[34] Groeneveld CS Fontugne J Cabel L Bernard-Pierrot I Radvanyi F Allory Y . Tertiary lymphoid structures marker CXCL13 is associated with better survival for patients with advanced-stage bladder cancer treated with immunotherapy. Eur J Cancer. (2021) 148:181–9. doi: 10.1016/j.ejca.2021.01.036, PMID: 33743486

[35] Hassan N Michaud E Mansure JJ Farag M Kool R Alessa M . The clinical relevance of tertiary lymphoid structures in assessing treatment response in muscle-invasive bladder cancer. Int J Radiat Oncol Biol Phys. (2026) 124:726–32. doi: 10.1016/j.ijrobp.2025.09.056, PMID: 41135713

[36] Yuan H Mao X Yan Y Huang R Zhang Q Zeng Y . Single-cell sequencing reveals the heterogeneity of B cells and tertiary lymphoid structures in muscle-invasive bladder cancer. J Trans Med. (2024) 22:48. doi: 10.1186/s12967-024-04860-1, PMID: 38216927 PMC10787393

[37] Ma G Jia H Zhang G Liang Y Dong X Fu G . Presence, subtypes, and prognostic significance of tertiary lymphoid structures in urothelial carcinoma of the bladder. Oncologist. (2023) 29:e248–58. doi: 10.1093/oncolo/oyad283, PMID: 37874923 PMC10836299

[38] Van Rijthoven M Obahor S Pagliarulo F Van Den Broek M Schraml P Moch H . Multi-resolution deep learning characterizes tertiary lymphoid structures and their prognostic relevance in solid tumors. Commun Med. (2024) 4:5. doi: 10.1038/s43856-023-00421-7, PMID: 38182879 PMC10770129

[39] An Y Sun J-X Xu M-Y Xu J-Z Ma S-Y Liu C-Q . Tertiary lymphoid structure patterns aid in identification of tumor microenvironment infiltration and selection of therapeutic agents in bladder cancer. Front Immunol. (2022) 13. doi: 10.3389/fimmu.2022.1049884, PMID: 36420257 PMC9676505

[40] Hamade A Li D Tyryshkin K Xu M Conseil G Yolmo P . Sex differences in the aging murine urinary bladder and influence on the tumor immune microenvironment of a carcinogen-induced model of bladder cancer. Biol Sex Differ. (2022) 13:19. doi: 10.1186/s13293-022-00428-0, PMID: 35505436 PMC9066862

[41] Ligon MM Liang B Lenger SM Parameswaran P Sutcliffe S Lowder JL . Bladder mucosal cystitis cystica lesions are tertiary lymphoid tissues that correlate with recurrent urinary tract infection frequency in postmenopausal women. J Urol. (2023) 209:928–36. doi: 10.1097/JU.0000000000003196, PMID: 36715657 PMC11463732

[42] Wang X Juncker-Jensen A Huang G Nagy ML Lu X Cheng L . Spatial relationship of tertiary lymphoid structures and tumor-associated neutrophils in bladder cancer and prognostic potential for anti-PD-L1 immunotherapy. Cancer Commun. (2024) 44:499–503. doi: 10.1002/cac2.12491, PMID: 37864307 PMC11024682

[43] Gupta S Gibb E Sonpavde GP Gupta S Maughan BL Agarwal N . Biomarker analysis and updated clinical follow-up from BLASST-1 (Bladder Cancer Signal Seeking Trial) of nivolumab, gemcitabine, and cisplatin in patients with muscle-invasive bladder cancer (MIBC) undergoing cystectomy. J Clin Oncol. (2022) 40:528. doi: 10.1200/JCO.2022.40.6_suppl.528

[44] Zhang L Zhang R Jin D Zhang T Shahatiaili A Zang J . Synergistic induction of tertiary lymphoid structures by chemoimmunotherapy in bladder cancer. Br J Cancer. (2024) 130:1221–31. doi: 10.1038/s41416-024-02598-7, PMID: 38332180 PMC10991273

[45] Van Dijk N Gil-Jimenez A Silina K Hendricksen K Smit LA De Feijter JM . Preoperative ipilimumab plus nivolumab in locoregionally advanced urothelial cancer: the NABUCCO trial. Nat Med. (2020) 26:1839–44. doi: 10.1038/s41591-020-1085-z, PMID: 33046870

[46] Gao J Navai N Alhalabi O Siefker-Radtke A Campbell MT Tidwell RS . Neoadjuvant PD-L1 plus CTLA-4 blockade in patients with cisplatin-ineligible operable high-risk urothelial carcinoma. Nat Med. (2020) 26:1845–51. doi: 10.1038/s41591-020-1086-y, PMID: 33046869 PMC9768836

[47] Cigliola A Moschini M Tateo V Mercinelli C Patanè D Crupi E . Perioperative sacituzumab govitecan (SG) alone or in combination with pembrolizumab (Pembro) for patients with muscle-invasive urothelial bladder cancer (MIBC): SURE-01/02 interim results. J Clin Oncol. (2024) 42:LBA4517–LBA4517. doi: 10.1200/JCO.2024.42.17_suppl.LBA4517

[48] Mercinelli C Moschini M Cigliola A Mattorre B Tateo V Basile G . First results of NURE-combo: A phase II study of neoadjuvant nivolumab and nab-paclitaxel, followed by postsurgical adjuvant nivolumab, for muscle-invasive bladder cancer. J Clin Oncol. (2024) 42:4196–205. doi: 10.1200/JCO.24.00576, PMID: 39241203

[49] Mercinelli C Basile G Proudfoot JA Jong JD Cigliola A Tateo V . Overall survival and biomarker results of NURE-Combo: A phase 2 study of neoadjuvant nivolumab (NIVO) and nab-paclitaxel (ABX) followed by postsurgical adjuvant NIVO in patients (pts) with muscle-invasive bladder cancer (MIBC). J Clin Oncol. (2025) 43:4594–4. doi: 10.1200/JCO.2025.43.16_suppl.4594

[50] Necchi A Jong JD Proudfoot JA Maiorano BA Cigliola A Tateo V . First results of SURE-02: A phase 2 study of neoadjuvant sacituzumab govitecan (SG) plus pembrolizumab (Pembro), followed by response-adapted bladder sparing and adjuvant pembro, in patients with muscle-invasive bladder cancer (MIBC). J Clin Oncol. (2025) 43:4518–8. doi: 10.1200/JCO.2025.43.16_suppl.4518

[51] Elizagaray ML BarraChina F Avenatti MC Bastepe I Chen A Odriozola A . Chronic inflammation drives epididymal tertiary lymphoid structure formation and autoimmune fertility disorders in mice. Nat Commun. (2025) 16:8742. doi: 10.1038/s41467-025-63514-y, PMID: 41034192 PMC12488923

[52] Magara T Nakamura M Nojiri Y Yoshimitsu M Kano S Kato H . Tumor immune microenvironment of cutaneous angiosarcoma with cancer testis antigens and the formation of tertiary lymphoid structures. Front Oncol. (2023) 13. doi: 10.3389/fonc.2023.1106434, PMID: 37081973 PMC10112511

[53] Bian Z Chen B Shi G Yuan H Zhou Y Jiang B . Single-cell landscape identified SERPINB9 as a key player contributing to stemness and metastasis in non-seminomas. Cell Death Dis. (2024) 15:812. doi: 10.1038/s41419-024-07220-5, PMID: 39528470 PMC11555415

[54] Xu W Ma C Liu W Anwaier A Tian X Shi G . Prognostic value, DNA variation and immunologic features of a tertiary lymphoid structure-related chemokine signature in clear cell renal cell carcinoma. Cancer Immunology Immunotherapy. (2022) 71:1923–35. doi: 10.1007/s00262-021-03123-y, PMID: 35043231 PMC10992571

[55] Teillaud J-L Houel A Panouillot M Riffard C Dieu-Nosjean M-C . Tertiary lymphoid structures in anticancer immunity. Nat Rev Cancer. (2024) 24:629–46. doi: 10.1038/s41568-024-00728-0, PMID: 39117919

[56] Liu C Cao J . The pivotal role of tertiary lymphoid structures in the tumor immune microenvironment. Front Oncol. (2025) 15. doi: 10.3389/fonc.2025.1616904, PMID: 40475020 PMC12137097

[57] Sobottka B Vetter V Banaei-Esfahani A Nowak M Lorch A Sirek A . Immune phenotype-genotype associations in primary clear cell renal cell carcinoma and matched metastatic tissue. Modern Pathol. (2024) 37:100558. doi: 10.1016/j.modpat.2024.100558, PMID: 38969270

[58] Tang H Su Z Huang Q Li Y Chen R Ban C . A model of tertiary lymphatic structure-related prognosis for penile squamous cell carcinoma. BMC Urol. (2024) 24:165. doi: 10.1186/s12894-024-01532-6, PMID: 39090582 PMC11295339

[59] Zavillová N Waldauf P Kendall Bártů M Čapka D Hojný J Prouzová Z . B lymphocytes and tertiary lymphoid structures have a prognostic impact on penile squamous cell carcinoma. J Pathol Clin Res. (2025) 11:e70059. doi: 10.1002/2056-4538.70059, PMID: 41235705 PMC12616500

[60] Hrudka J Prouzová Z Kendall Bártů M Hojný J Čapka D Zavillová N . Immune cell infiltration, tumour budding, and the p53 expression pattern are important predictors in penile squamous cell carcinoma: a retrospective study of 152 cases. Pathology. (2023) 55:637–49. doi: 10.1016/j.pathol.2023.03.010, PMID: 37316384

[61] Xue T Deng C Liu J Yan R Li J Hu X . Spatial transcriptional dynamics of CD74^+^ B cells in tertiary lymphoid structures drive immune evolution in penile squamous cell carcinoma. Adv Sci (Weinh). (2025) 12:e09742. doi: 10.1002/advs.202509742, PMID: 41111459 PMC12667480

[62] Touloupidis S Zisimopoulos A Giannakopoulos S Papatsoris AG Kalaitzis C Thanos A . Clinical usage of the squamous cell carcinoma antigen in patients with penile cancer. Int J Urol. (2007) 14:174–6. doi: 10.1111/j.1442-2042.2007.01694.x, PMID: 17302580

[63] Li K Sun J Wei X Wu G Wang F Fan C . Prognostic value of lymphovascular invasion in patients with squamous cell carcinoma of the penis following surgery. BMC Cancer. (2019) 19:476. doi: 10.1186/s12885-019-5714-1, PMID: 31113402 PMC6528249

[64] Yi X Lu H Li W Tang Y . Venous thrombosis, multiple carcinomatous foci and differences in metastatic pathways of penile carcinoma. Oncol Lett. (2023) 25:88. doi: 10.3892/ol.2023.13674, PMID: 36817041 PMC9932005

[65] Czajkowski M Wierzbicki PM Dolny M Matuszewski M Hakenberg OW . Inflammation in penile squamous cell carcinoma: A comprehensive review. Int J Mol Sci. (2025) 26. doi: 10.3390/ijms26062785, PMID: 40141426 PMC11943298

[66] Miyagi H Yu X Peak T Dhillon J Le C Wang X . Progressive T cell exhaustion and predominance of aging tissue associated macrophages with advancing disease stage in penile squamous cell carcinoma. Sci Rep. (2025) 15:7703. doi: 10.1038/s41598-025-89760-0, PMID: 40044748 PMC11882776

[67] Guimarães SJA Vale AAM Rocha MCB Butarelli ALA Da Silva JM De Deus AJS . Human papillomavirus infection affects the immune microenvironment and antigen presentation in penile cancer. Front Oncol. (2024) 14:1463445. doi: 10.3389/fonc.2024.1463445, PMID: 39493451 PMC11527599

[68] Tan X Wang Y Yu Y Zheng R Li J Chen S . Neutrophil-to-lymphocyte ratio predicts a poor prognosis for penile cancer with an immunosuppressive tumor microenvironment. Front Immunol. (2025) 16:1568825. doi: 10.3389/fimmu.2025.1568825, PMID: 40308599 PMC12041217

[69] Mumba C Mwale NK Mapulanga V Ngalamika O . CD4 and CD8 T-cell lymphocytes from penile squamous cell carcinoma tumors are more differentiated with higher PD-1 expression compared to lymphocytes in peripheral circulation. Immunol Lett. (2026) 277:107099. doi: 10.1016/j.imlet.2025.107099, PMID: 41045992 PMC12547531

[70] Ionescu F Nguyen J Segura CM Paravathaneni M Grass GD Johnstone P . Multiplex immunofluorescence captures progressive immune exhaustion with advancing penile squamous cell cancer stage. Cancers (Basel). (2024) 16. doi: 10.3390/cancers16020303, PMID: 38275860 PMC10814242

[71] Azhar F Sachdeva A Hart CA Brown MD Sangar V Parnham A . Differential expression of PDL-1 and tumour-associated macrophages in N0 and N+ penile cancer. J Clin Oncol. (2023) 41:12–2. doi: 10.1200/JCO.2023.41.6_suppl.12

[72] Ottenhof SR Djajadiningrat RS Thygesen HH Jakobs PJ Jóźwiak K Heeren AM . The prognostic value of immune factors in the tumor microenvironment of penile squamous cell carcinoma. Front Immunol. (2018) 9:1253. doi: 10.3389/fimmu.2018.01253, PMID: 29942303 PMC6004546

[73] Joshi VB Spiess PE Necchi A Pettaway CA Chahoud J . Immune-based therapies in penile cancer. Nat Rev Urol. (2022) 19:457–74. doi: 10.1038/s41585-022-00617-x, PMID: 35851333

[74] Tang Y Hu X Wu K Li X . Immune landscape and immunotherapy for penile cancer. Front Immunol. (2022) 13:1055235. doi: 10.3389/fimmu.2022.1055235, PMID: 36524123 PMC9745054

[75] Winkelmann R Becker N Leichner R Wild PJ Demes M Banek S . Gene expression profiling of the peritumoral immune cell infiltrate of penile squamous cell carcinomas. Int J Mol Sci. (2024) 25. doi: 10.3390/ijms252212142, PMID: 39596210 PMC11594387

[76] Chen Z Wang X Jin Z Li B Jiang D Wang Y . Deep learning on tertiary lymphoid structures in hematoxylin-eosin predicts cancer prognosis and immunotherapy response. NPJ Precis Oncol. (2024) 8:73. doi: 10.1038/s41698-024-00579-w, PMID: 38519580 PMC10959936

[77] Hammouda K Tokuyama N Corredor G Pathak T Dakarapu R Genega E . AI-informed computational pathology classifier predicts outcomes across treatment modalities in muscle-invasive urothelial carcinoma. Cancer Lett. (2025) 634:218059. doi: 10.1016/j.canlet.2025.218059, PMID: 40998194 PMC12496026

[78] Hammouda K Corredor G Pathak T Mian OY Pavicic PG Diaz-Montero CM . Correlation of artificial intelligence (AI)-based spatial characteristics of tumor-infiltrating lymphocytes outcomes with immune checkpoint inhibitors (ICIs) in patients (pts) with metastatic urothelial cancer (mUC). In: 2024 ASCO genitourinary cancers symposium. ASCO Publications: American Society of Clinical Oncology (2024). doi: 10.1200/JCO.2024.42.4_suppl.691

[79] Helmink BA Reddy SM Gao J Zhang S Basar R Thakur R . B cells and tertiary lymphoid structures promote immunotherapy response. Nature. (2020) 577:549–55. doi: 10.1038/s41586-019-1922-8, PMID: 31942075 PMC8762581

[80] Su GL Zhang MJ Li H Sun ZJ . Dissecting tertiary lymphoid structures in cancer: maturation, localization and density. Theranostics. (2025) 15:9459–85. doi: 10.7150/thno.113940, PMID: 41041059 PMC12486156

[81] Sautès-Fridman C Petitprez F Calderaro J Fridman WH . Tertiary lymphoid structures in the era of cancer immunotherapy. Nat Rev Cancer. (2019) 19:307–25. doi: 10.1038/s41568-019-0144-6, PMID: 31092904

[82] Colbeck EJ Ager A Gallimore A Jones GW . Tertiary lymphoid structures in cancer: drivers of antitumor immunity, immunosuppression, or bystander sentinels in disease? Front Immunol. (2017) 8. doi: 10.3389/fimmu.2017.01830, PMID: 29312327 PMC5742143

